# Enrichment of the Antibiotic Resistance Gene *tet*(L) in an Alkaline Soil Fertilized With Plant Derived Organic Manure

**DOI:** 10.3389/fmicb.2018.01140

**Published:** 2018-05-31

**Authors:** Shuang Peng, Jan Dolfing, Youzhi Feng, Yiming Wang, Xiangui Lin

**Affiliations:** ^1^Key Laboratory of Soil Environment and Pollution Remediation, Institute of Soil Science, Chinese Academy of Sciences, Nanjing, China; ^2^College of Environment and Ecology, Jiangsu Open University, Nanjing, China; ^3^School of Engineering, Newcastle University, Newcastle upon Tyne, United Kingdom

**Keywords:** antibiotic resistance genes, class 1 integron, organic manure, archived soil, real-time PCR

## Abstract

Fifteen antibiotic resistance genes (ARGs) and *intI1*, a gene involved in horizontal gene transfer (HGT) of ARGs, were quantified in three different soil samples from a 22 year old field experiment that had received inorganic fertilizer (NPK), organic manure (OM; a mixture of wheat straw, soybean oil cake and cotton cake), and control fields that had received no fertilizer and manure (CK). *Tet*(L) was the most abundant ARG in OM, which also contained considerable levels of *intI1*. Molecular analysis of yearly collected archived soils over the past 22 years showed that *tet*(L) and *intI1* were higher in OM soils than in NPK soils. The relative abundance of *tet*(L) was essentially constant during these years, while the level of *intI1* in OM soils decreased over time. The main genotype of *tet*(L) was the same in archived and in fresh soil, OM, and irrigation water. Phylogenetic analysis of the 16S rRNA genes of tetracycline-resistant bacteria (TRB) isolates indicated that the *Firmucutes* carrying *tet*(L) in OM were similar to those in the OM soil, suggesting that OM transferred TRB into the OM soils where they survived. Almost all of the TRB isolated from OM carried *tet*(L) and belonged to the *Firmicutes*. Survival of bacteria from the organic manure that carried *tet*(L) may be the cause of the increased level of *tet*(L) in OM soil.

## Introduction

Antibiotic resistance genes (ARGs) in the environment are posing a global threat to public health (Pruden et al., 2013). Antibiotic misuse and over-prescription have been regarded as driving forces promoting the selection and dissemination of resistance (D'Costa et al., 2007). Horizontal gene transfer (HGT) of mobile genetic elements harboring ARGs substantially increases the epidemiological risks associated with antibiotic resistance, because these elements can be readily transferred between bacteria on both an inter- and intra-species level or bi-directionally from non-pathogenic to pathogenic bacteria (Forsberg et al., 2012; Cytryn, 2013).

Previous studies have focused on the effect of animal manure on antibiotic resistance in agricultural soils, showing that use of animal manure as fertilizer increases resistance of cultivable soil microorganism to tetracycline and sulfonamide antibiotics (Sengeløv et al., 2003; Byrne-Bailey et al., 2009). The occurrence, abundance, and transferability of ARGs in soil especially increased

after field application of manure from antibiotic-treated animals (Jechalke et al., 2013; Kyselková et al., 2013; Zhu et al., 2013; Peng et al., 2015, 2017). In addition, it has been reported that manure from cattle that had not been treated with antibiotics can also lead to increased populations of resident soil bacteria harboring β-lactam resistance genes (Udikovic-Kolic et al., 2014).

Application of organic manure (OM) from plants rather than animals usually increases soil microbial biomass (Chu et al., 2007) and crop yield (Chen et al., 2010), and ameliorates soil properties, both abiotically, and biotically (Feng et al., 2015). OM fertilization has a large influence on bacterial community composition, larger than fertilization with chemical fertilizers (Feng et al., 2015). A previous study has reported that the different effects of pig manure with that of chemical fertilizers and straw on the soil resistome was accompanied by a change in the bacterial community (Liu et al., 2017). OM from plants is quite different from straw and animal manure, both chemically and microbiologically. In addition, OM is frequently composted and fungi and bacteria are known to play important role during the organic waste composting process (Neher et al., 2013; Gu et al., 2017). The present study was directed toward investigating the impact of long-term application of OM on the soil resistome and the main factor directing the impact.

Archived soil samples are a very valuable resource for microbial ecological studies (Dolfing et al., 2004; Cary and Fierer, 2014; Dolfing and Feng, 2015). Knapp et al. (2010) have assayed DNA extracted from dried archived soils, and found that the presence of ARGs in agricultural soils has substantially increased since 1940, concomitant with the increase in industrial antibiotic production. Graham et al. (2016) analyzed soils archived since 1923 and found that “total” β-lactam ARG levels were significantly higher in manured soils than in inorganic fertilized soils after 1940; in addition, these authors showed that when non-therapeutic antibiotic use was banned in Denmark, the levels of the extended-spectrum β-lactamase gene bla_CTX−M_ declined in manured soils. Dolfing et al. (2004) showed that air-dried soils stored for extended periods of time is feasible for application of molecular techniques, generating valuable information on bacterial community changes due to agricultural practice, even though drying and storage can affect microbial biomass and activity (De Nobili et al., 2006). Tzeneva et al. (2009) also found that soil drying and storage affected the detectable community structure in their samples, but did not materially impair their capacity to identify the effect of soil parameters studied in long-term grassland experiments. Given the high scientific value of archived soil samples, it is relevant to evaluate the effect of long-term manure application on the abundance of ARGs with archived samples.

Based on the above considerations, ARG abundance in fresh soil samples from a 22 years field fertilizer experiment, including non-animal waste OM fertilization and chemical fertilizations with NPK (nitrogen-phosphorus-potassium) as well as a control without fertilization, were analyzed by qPCR. Furthermore, archived soils yearly collected from 1989 onwards were analyzed to assess the change of the main ARG over the past 20 years. To compare the historical effect of non-animal organic manure vs. inorganic fertilizer, phylogenetic analysis of the main ARG was also conducted to obtain information on the potential source of this gene. The resistant bacteria carried with the main ARG was isolated and identified to further confirm the source of main ARG. Present study comprehensively investigated the influence of OM from plant on soil ARGs and the results also can help us better understand the mechanism of the influence on soil resistome caused by animal manure.

## Materials and methods

### Description of the long-term experiment

The long-term field fertilizer experiment was conducted at Fengqiu Agro-ecological Experimental Station, Chinese Academy of Sciences, Fengqiu County, Henan Province, China (35°00′N, 114°24′E). The soil was a sandy loam (alluvial-aquic) soil with alkaline characteristics. Historical soil samples were acquired from Feng et al. (2015), soil physicochemical properties were described in detail in that article. Winter wheat (*Triticum aestivum L*.) and summer maize (*Zea mays L*.) were rotated planted, and agronomic practice was not substantially changed for more than 20 years. At the beginning of the experiment in 1989, the soil contained 5.83 g kg^−1^ of organic matter, 0.45 g total N kg^−1^, 0.50 g total P kg^−1^, and 18.6 g total K kg^−1^ with a pH of 8.65. Three treatments with four replicates in completely randomized blocks were established. were selected for analysis in this study: (1) OM: applied with organic manure (P and K were supplemented as chemical fertilizers for the same amount of nutrients as other treatments); (2) NPK: balanced fertilizations with NPK; (3) Control: no fertilization. The composition of OM was constant during these years; OM consisted of wheat straw, soybean oil cake and cotton cake at a ratio of 100:40:45, as described in detail by Meng et al. (2005) and Gong et al. (2009).

### Sample collection and determination

Fresh soil samples were collected on 18 September 2011 (at maize harvesting stage) from 16 points at a depth of 0–15 cm from each field as described in Feng et al. (2015). Sub-samples were air dried and kept at room temperature. Fresh samples were kept at −80°C until further use. A series of soil samples from 1989 to 2009 under NPK, OM, and control fertilization regimes was collected as described in Feng et al. (2015).

### Soil and organic manure DNA extraction

DNA was extracted from soil using the FastDNA® SPIN Kit for soil (MP Biomedicals) according to the manufacturer's protocol. DNA of OM was extracted using the FastDNA® SPIN Kit for feces samples. The extracted DNA was dissolved in 50-μL of TES buffer, quantified by a microspectrophotometry (NanoDrop ND-1000, NanoDrop Technologies, Wilmington, DE), and stored at −20°C until use. The DNA samples were diluted to 1:10 and 1:50 to avoid inhibitors to the PCR reaction.

### Quantification of ARGs, 16S-rRNA, and class I integrons

qPCR analysis was used to determine the abundance of ARGs and class I integrons (*intI1*) by C1000™ Thermal Cycler equipped with the CFX96 Touch™ Real-Time PCR Detection System (Bio-Rad, USA). All qPCR assays were performed in triplicate using the SYBR *Premix Ex Taq*™ *Kit* (TaKaRa) with the primer sets shown in Table S1. A 10-fold dilution series of plasmids carrying each targeted ARG was made to generate a six-point calibration curve for qPCR. The abundance of ARGs per microliter of plasmid solution was calculated according to Zhang et al. (2009). All samples were assayed in duplicate, as were the standards, and positive and negative controls. Bacterial 16S rRNA genes were also quantified as described by Biddle et al. (2008), so that ARGs or *intI1* abundance could be normalized to the total bacterial 16S rRNA gene counts.

### Sequencing and phylogenetic analysis of *tet*(L)

Archived OM soil samples of 1991, 1993, 1997, 1999, 2001, 2003, 2005, 2007, 2009, and 2011, as well as fresh NPK soil and OM soil from 2011, fresh OM, irrigation water were selected for phylogenetic analysis of *tet*(L). *tet*(L) gene sequencing was performed by Tianhao Genomics Institute (Shanghai, China) using the SMRT cell PACBIO RS II platform with primers *tet*(L)-F (5′-GTTGCGCGCTATATTCCAAA-3′) and *tet*(L)-R (5′-TTAAGCAAACTCATTCCAGC-3′) (You et al., 2012). The number of clean reads of every sample was more than 1900. Sequences were clustered into an genotype at a level of 99% sequence similarity, and one representative sequence was selected for subsequent similarity analysis online using BLASTn and for phylogenetic tree construction. The neighbor joining trees were constructed using MEGA version 5.1.

### Isolation and identification of tetracycline-resistant bacteria carrying *tet*(L)

Tetracycline-resistant bacteria (TRB) and culturable bacteria were counted in fresh OM, fresh soils from the NPK and OM treated fields, and in the archived OM soil from 1989 and 2011. Soil samples were suspended in saline and plated onto R2A agar (Popowska et al., 2012) medium supplemented with and without antibiotics. Each of the antibiotic medium plates contained 8 μg/mL of tetracycline, based on Sengeløv et al. (2003). The plates were incubated at room temperature for 3–4 days. Twenty strains of TRB were randomly isolated from each soil sample. The purified strains were stored at 4°C on antibiotic agar plates.

The colony PCR method was used for the amplification of 16S rRNA and *tet*(L) genes. Amplification of 16S rRNA genes was performed with primers 27F (5′-AGAGTTTGATCCTGGCTCAG-3′) and 1492R (5′-GGTTACCTTGTTACGACTT-3′) under the conditions published by Popowska et al. (2012). Subsequently the isolated TRB were analyzed for presence of *tet*(L). The 16S rRNA gene PCR products of those bacteria carrying *tet*(L) were selected and ligated into a pEASY-T3 vector (TransGen Biotech, Beijing) and then cloned into a Trans1-T1 phage resistant chemically competent cell (TransGen Biotech, Beijing). Positive clones were sequenced, and identification to the species level was performed. Bacteria sharing ≥99% identity with 16S rRNA gene sequences in EzBioCloud and Genbank were identified to the species level; those with 95–98.9% identity were identified to the genus level (Popowska et al., 2012).

### Heavy metal analysis

Eight typical heavy metals, viz. Hg, As, Cd, Cr, Pb, Cu, Zn, and Ni, were quantified using the method developed by Lu (2000). These metals are found frequently in the environmena as a result of human activities or are commonly used as additives in livestock feed (Chen et al., 2009; Ji et al., 2012). Samples were digested in teflon crucibles at 170–210. Hg and As levels in soils was quantified by an atomic fluorescence spectrophotometer AFS-8120 as described by GB/T 22105-2008. The other six heavy metals were quantified using high performance liquid chromatography inductively coupled plasma-mass spectroscopy (HPLC-ICP-MS).

### Statistical analysis

Statistical analysis was carried out using the software package SPSS 17.0 for Windows (SPSS Inc., USA). Figures were plotted with OriginPro 8.0 (OriginLab Corporation, USA). Mean separation was assessed by ANOVA. Duncan's multiple range test was used to evaluate the significance of differences between samples. *P* < 0.05 was considered statistically significant. Correlations between the relative abundance of *tet*(L) and *intI1* or heavy metal content (Pearson's correlation coefficient) were calculated with SPSS 17.0.

## Results

### Abundance of ARGs in OM, irrigation water, and fresh soil samples

**Figure 1:**
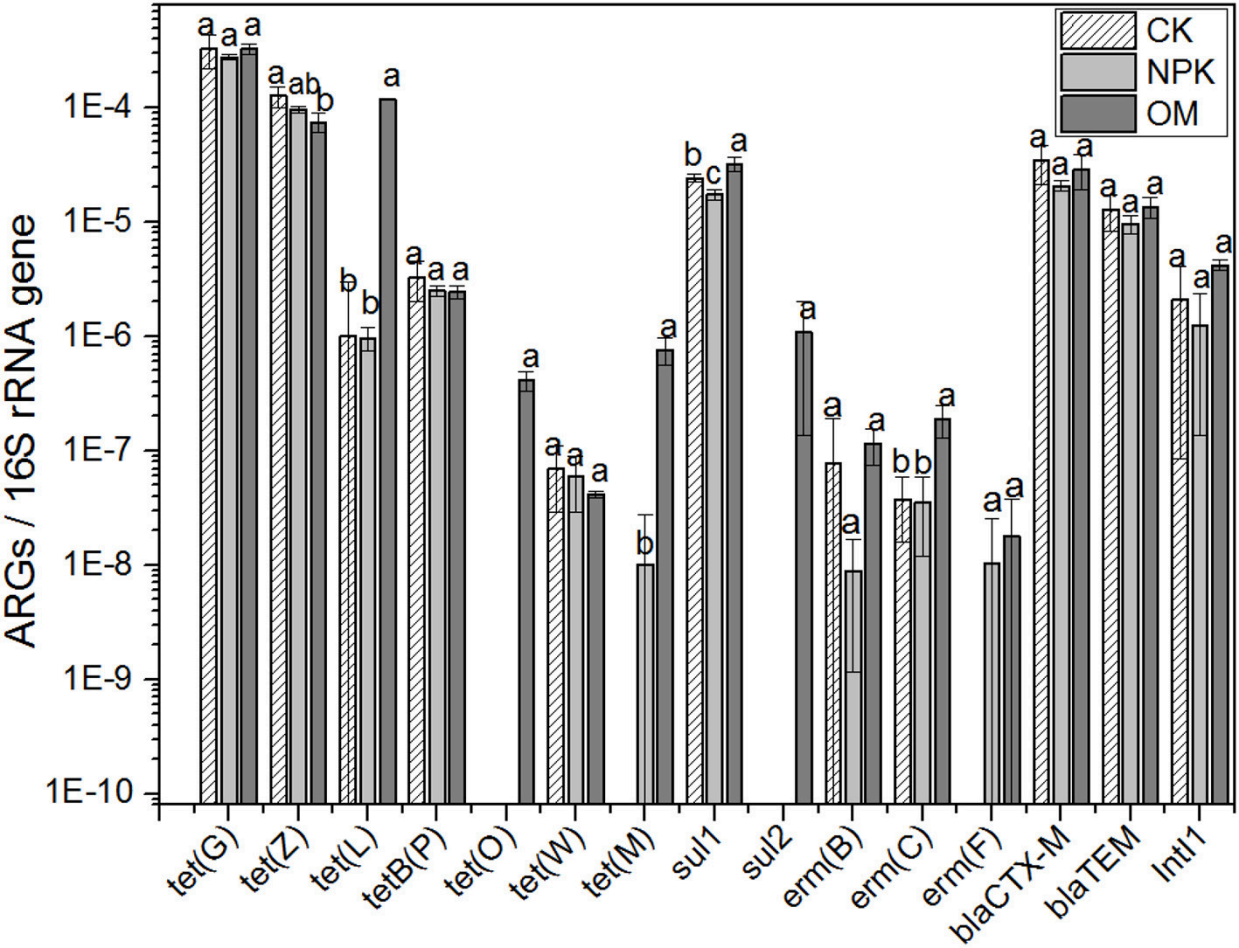
The relative abundance of ARGs in fresh soil fertilized with OM or NPK and CK.

The distribution of 15 ARGs and two *intI* genes, normalized to 16S rRNA gene copies, in OM and irrigation water was evaluated by real-time PCR. ARGs are abundant in OM. The most abundant ARG was *tet*(L) (Table 1), which accounted for 67.49% of the total relative abundance of the 15 ARGs, followed by *erm*(C), *sul1*, and *sul2*. Irrigation water also contained considerable amounts of ARGs, of which the most abundant ARGs are *erm*(C), *sul2, sul1*, and *tet*(G) (Table 1). In the fresh soil samples collected in 2011, the relative abundances of *tet*(L), *tet*(M), *sul1*, and *erm*(C) were significantly increased after continuous application of OM for 22 years; *tet*(O) and *sul2* had also increased to a detectable level (Figure 1). Among these ARGs, *tet*(L) had increased the most: the relative abundance of *tet*(L) in OM soil was 120 times more than that in NPK soil, followed by *tet*(M), which was increased by 74.5 times. In our previous studies, we found that *tet*(L) also had increased considerably in soil after long-term fertilization with animal manure (Peng et al., 2015, 2017); therefore this gene was selected for comprehensive analysis in this study.

**Table 1:** Abundance of ARGs and 16S rRNA gene in organic manure (OM) and irrigation water.

	**OM**		**Irrigation water**	
**Gene target**	**Copies/g FW manure**	**Relative abundance**	**Copies/L**	**Relative abundance**
*tet*(G)	1.67E+07	4.04E−05	3.21E+05	3.10E−05
*tet*(L)	1.40E+09	3.39E−03	9.05E+04	8.72E−06
*tet*(Z)	2.03E+06	4.92E−06	6.36E+03	6.13E−07
*tetB*(P)	9.26E+04	2.24E−07	1.67E+04	1.61E−06
*tet*(O)	1.64E+07	3.96E−05	6.74E+02	6.49E−08
*tet*(W)	9.89E+07	2.39E−04	5.21E+02	5.02E−08
*tet*(M)	1.79E+07	4.32E−05	3.27E+03	3.15E−07
*sul1*	1.60E+08	3.88E−04	4.05E+05	3.91E−05
*sul2*	1.30E+08	3.14E−04	1.41E+06	1.36E−04
*sul3*	7.23E+03	1.75E−08	8.00E+01	7.71E−09
*erm*(B)	1.35E+06	3.28E−06	6.66E+04	6.42E−06
*erm*(C)	2.16E+08	5.22E−04	4.26E+06	4.11E−04
*erm*(F)	1.59E+07	3.84E−05	1.35E+04	1.30E−06
*bla*_CTX−M_	6.11E+04	1.48E−07	2.00E+02	1.92E−08
*bla*_TEM_	4.39E+04	1.06E−07	2.03E+02	1.95E−08
*intI1*	1.60E+08	3.87E−04	5.03E+04	4.85E−06
*intI2*	6.89E+06	1.67E−05	4.53E+02	4.37E−08
16S rRNA	4.13E+11		1.04E+10	

### Change of the *tet*(L) abundance after soils were air dried

**Figure 2:**
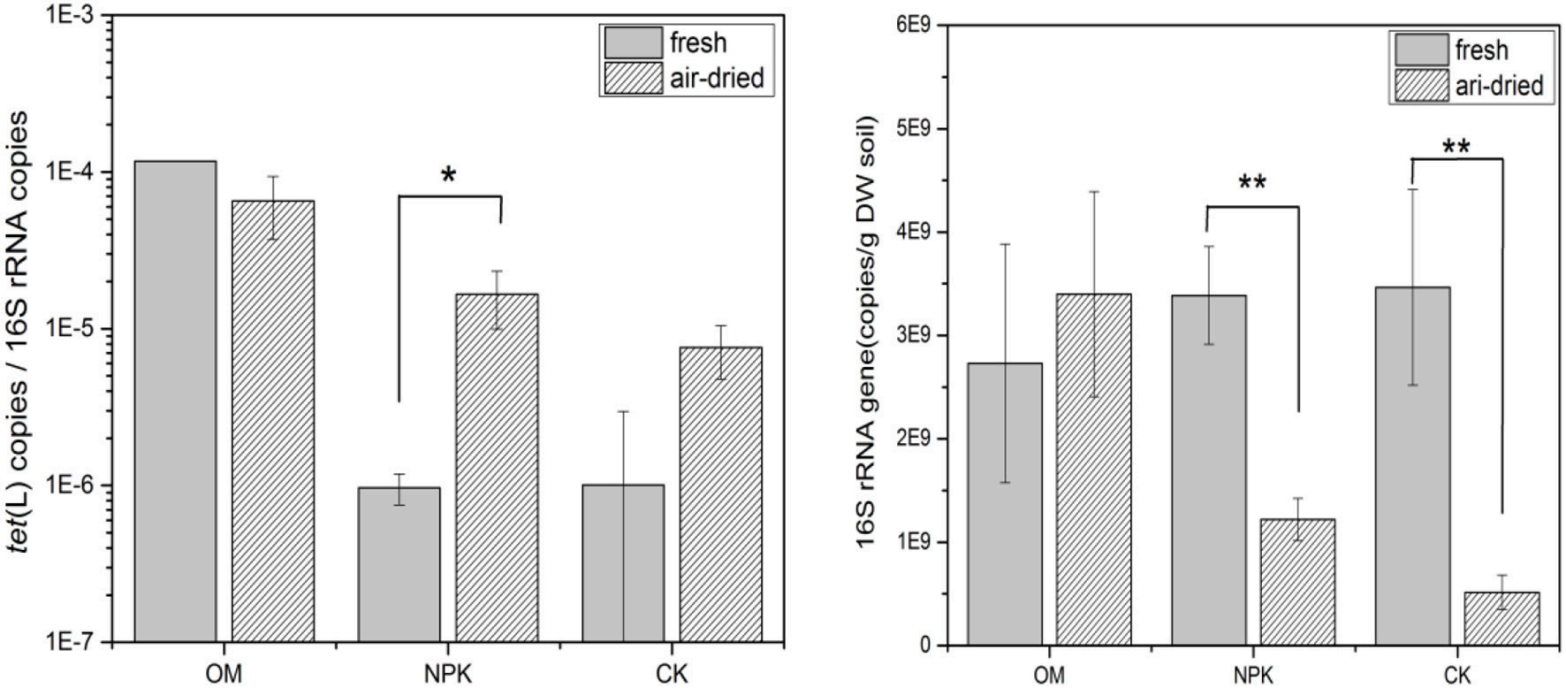
The relative abundance of *tet*(L) and the absolute abundance of the 16S rRNA gene in fresh or air-dried soil fertilized with OM or NPK and CK. ^*^ and ^**^ indicate statistically significant differences at *P* < 0.05 and *P* < 0.01, respectively.

We compared the relative abundance of *tet*(L) in fresh soils and air-dried soils, and found that *tet*(L) was increased in NPK and CK soils after the soils was air-dried, but not significantly changed in OM soil (Figure 2). Simultaneously, the abundance of the 16S rRNA gene was significantly reduced in NPK and CK after these soils were air-dried, while its abundance in the soil sampled from the fields fertilized with OM was not measurably changed (Figure 2). The abundance of *tet*(L) was not influenced by air-drying in OM soil.

### Abundance of *tet*(L) *and intI1*, and concentrations of heavy metals in yearly archived soil

**Figure 3:**
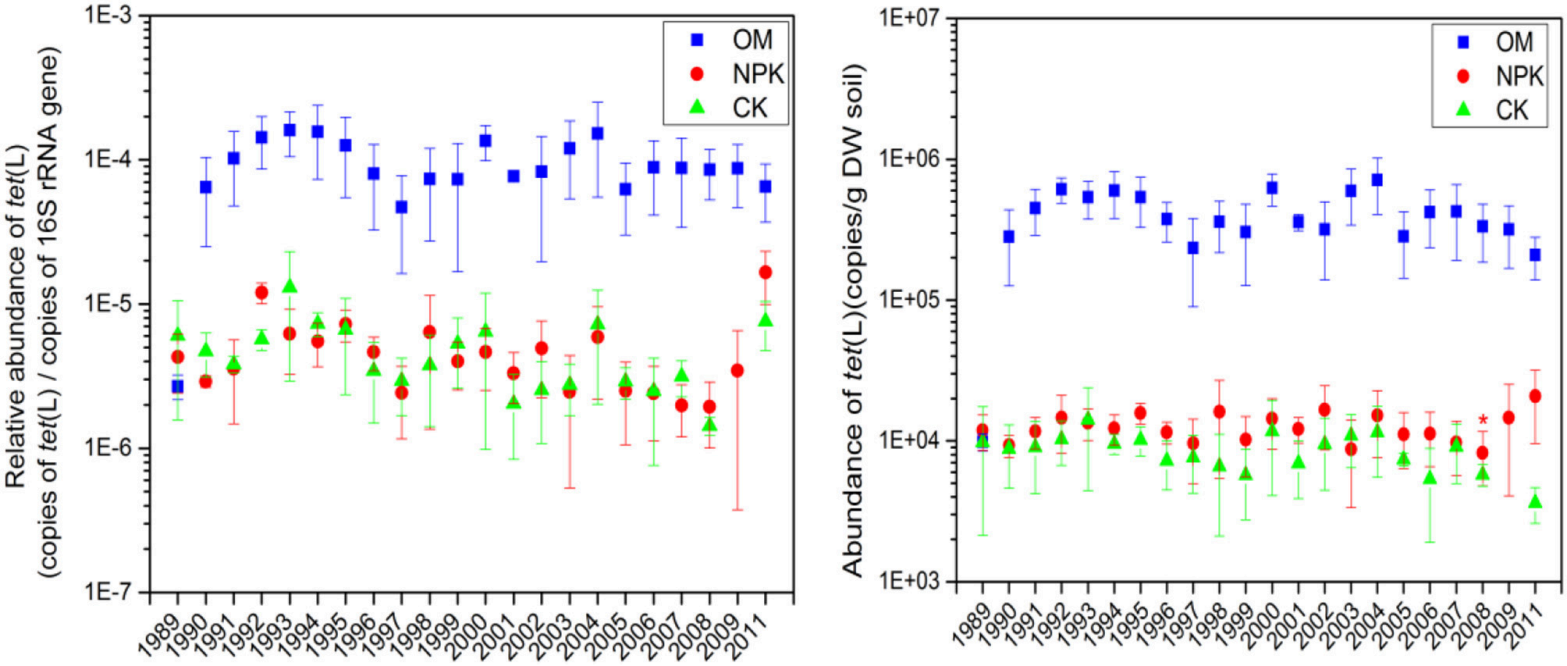
Changes in the abundance of the *tet*(L) in different fertilizer treated archived soils.

The relative abundance of *tet*(L) in CK, NPK, and OM treated soils fluctuated over time (Figure 3), but there was no distinct difference in relative abundance of the *tet*(L) between CK and NPK soils. *tet*(L) was markedly higher in OM soils than in NPK and CK soils (Figure 1), and this difference was consistent over 20 years (Figure 3). ANOVA with repeated measures revealed that these three treatments had no significant effect on the absolute abundance of *tet*(L) in the 22-years time series measurements.

**Figure 4:**
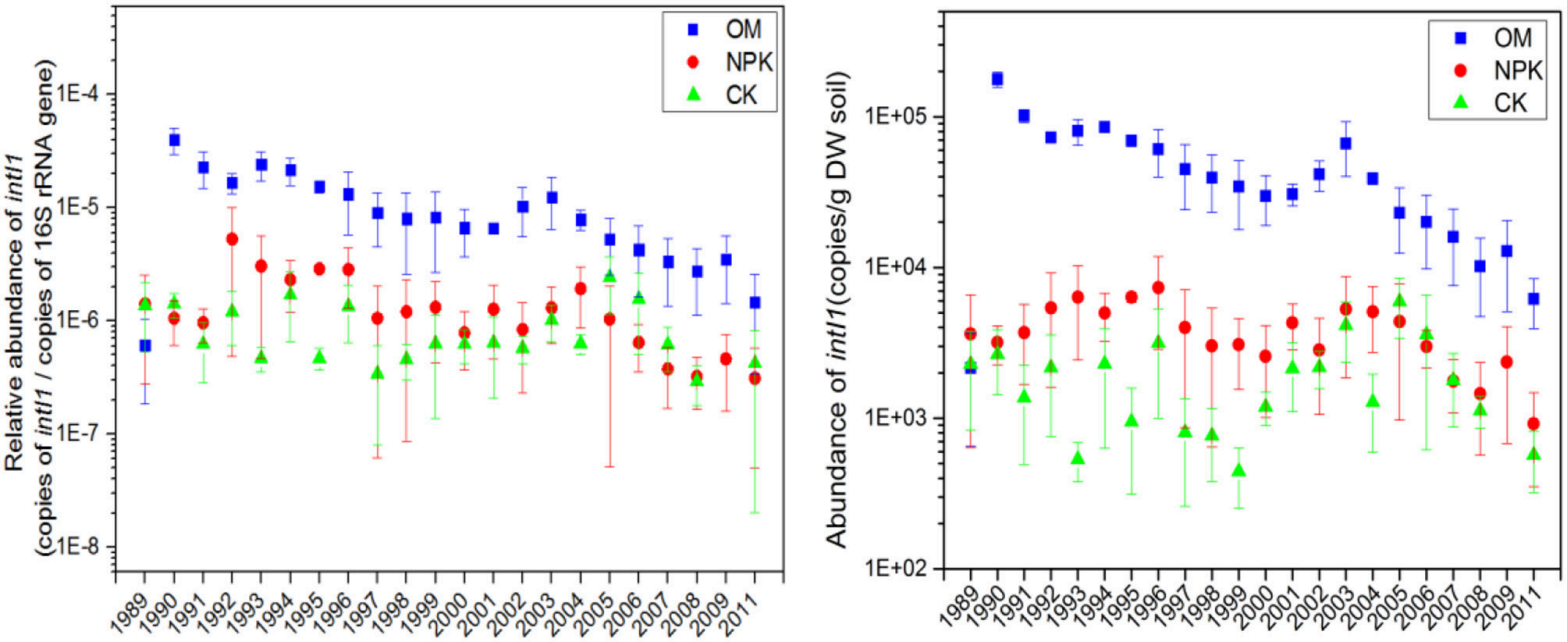
The variation trend in the abundance of the *intI1* gene in different fertilizer treated archived soils.

The abundance of *intI1* was at a very low level in those soils that were not exposed to OM; however, the relative abundance of *intI1* was higher in OM soils (Figure 4). Both the absolute and the relative *intI1* abundance decreased over time (Figure 4). A repeated measures one-way ANOVA revealed that all three treatments exerted no significant influence on the abundance of *intI1* among the 22 times measurement. *IntI2* was below the detection limit. *intI1* was positively correlated with *tet*(L) (*r* = 0.694, *P* < 0.01, Figure S1).

The heavy metal concentrations (Cu, As, Zn, Cr, Ni, Pb, As, and Hg) in archived soils of 1989, 1996, 2002, 2008, and 2011 did not significantly change over time (Table S3). Table S4 gives a summary of the correlation analyses among the detected heavy metals and *tet*(L), *intI1* in the soils. Cu and Pb were positively correlated with *tet*(L).

### The main *tet*(L) genotype in OM and OM soil

**Figure 5:**
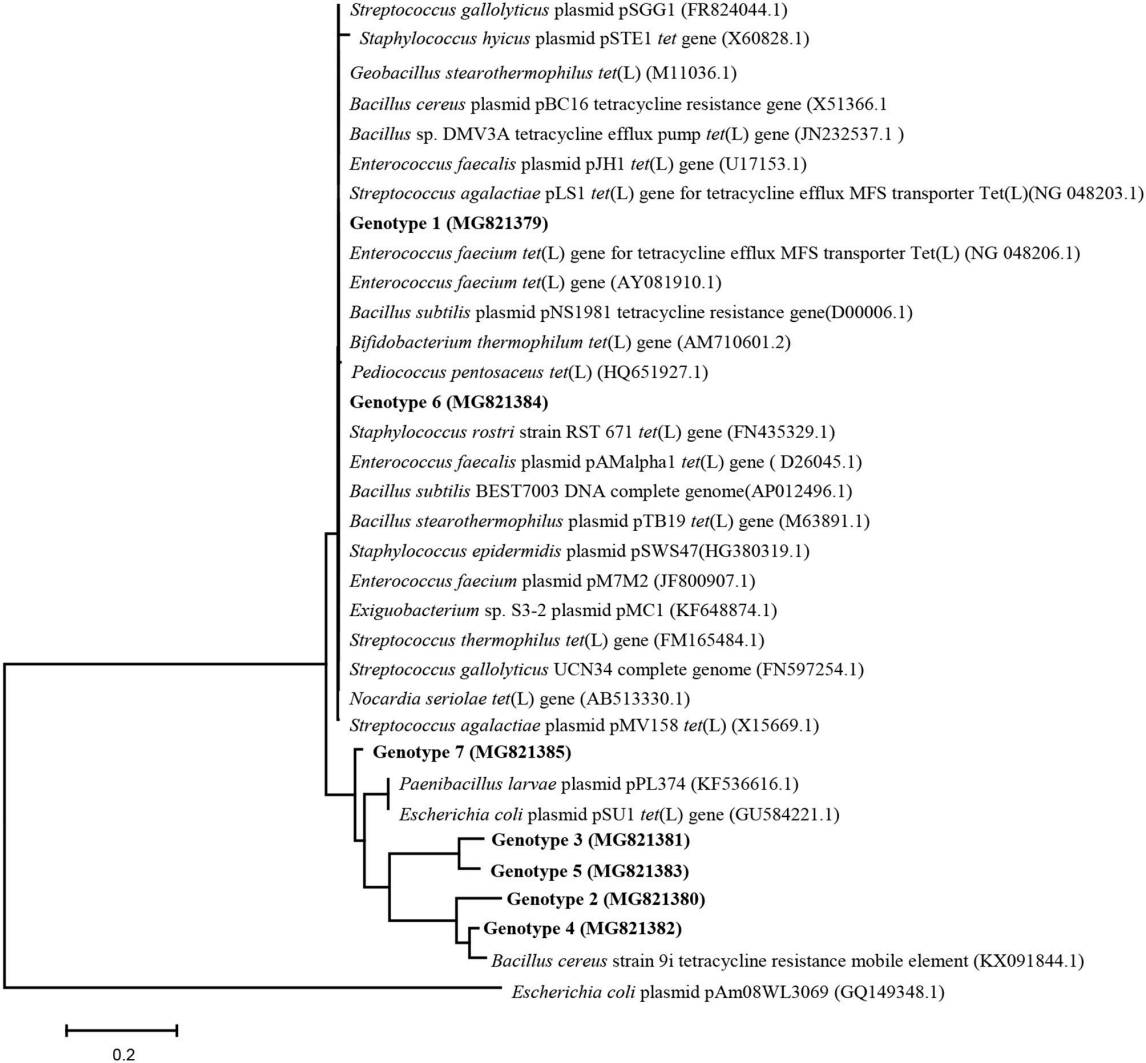
Neighbor-joining phylogenetic analysis of genotypes genotype 1~7 of *tet*(L) displayed in bold font. The sequences in GenBank that were most similar to genotype1–genotype 7 were retrieved for phylogenetic tree construction. The tree was constructed using MEGA version 5.1, and bootstrap analysis with 500 replicates was used to evaluate the significance of the nodes.

44896 *tet*(L) sequences were obtained and grouped into 230 genotypes under 99% identity. There were seven genotypes (genotype 1–genotype 7) containing more than two sequences, comprising 99.5% of the total library. The sequences of the seven genotypes have been deposited in GenBank under the accession number of MG821379–MG821385. Genotype1 was the main *tet*(L) genotype in all of the samples conducted for phylogenetic analysis, more than 97% sequences of every sample were grouped into genotype 1 (Figure S2). BLAST searches of the GenBank database confirmed that genotype 1 matched known *tet*(L) genes (Figure 5), with sequence identities of 100%. This most common *tet*(L) type was identical to the sequences of *tet*(L) found in plasmids and chromosomes of different Gram-positive bacteria.

### Isolation and identification of TRB carrying *tet*(L)

In order to find which species of ARB were transmitted into soil by OM and caused the difference of *tet*(L) abundance between OM soils and NPK soils, approximately 280 TRB were isolated from fresh OM, fresh soils of OM and NPK, and from the archived soil samples collected in 1989 and 2011 from OM treatment (Table 2). Fertilization with OM increased the number of TRB carrying *tet*(L). There were more diverse types of TRB carrying *tet*(L) in fresh soils fertilized with OM than in fresh soils fertilized with NPK (Table 2). Thirtythree of the 60 TRB (55%) isolated from the archived soils of 1989 carried *tet*(L); in the archived soils of 2011, the percentage of TRB carrying *tet*(L) increased to 91.7%. Almost all the TRB isolated from OM were carrying *tet*(L); they were mainly *Bacillus* sp., *Lysinibacillus* sp., *Solibacillus* sp*., Rummeliibacillus* sp., and *Sporosarcina* sp., all belonging to the order of the *Bacillales*.

**Table 2:** Tetracycline resistant bacteria isolated from OM and the fresh or archived soil.

	**OM**	**Archived OM soil from 2011**	**Archived OM soil from 1989**	**Fresh OM soil**	**Fresh NPK soil**
Culturable bacteria (CFU/g)	3.93 ± 0.24(×10^8^)	1.12 ± 0.08(×10^8^)	0.31 ± 0.11(×10^8^)	2.79 ± 0.81(×10^8^)	0.46 ± 0.03(×10^8^)
TRB (CFU/g)	3.63 ± 1.04(×10^6^)	1.07 ± 0.78(×10^6^)	0.02 ± 0.01(×10^6^)	5.51 ± 3.11(×10^6^)	2.04 ± 0.68(×10^6^)
NO. of isolated TRB	42	60	60	60	62
TRB carrying *tet*(L)	41	55	33	11	2
Best phylogenetic match (No. of isolates)	*Bacillus toyonensis* (1)	*Bacillus proteolyticus*(2)	*Bacillus paramycoides*(2)	*Lysinibacillus fusiformis* (1)	*Lysinibacillus macroides*(2)
	*Bacillus circulans*(10)	*Bacillus circulans*(5)	*Bacillus cereus*(5)	*Lysinibacillus sphaericus*.(2)	
	*Bacillus galactosidilyticus*(2)	*Bacillus firmus*(1)	*Bacillus velezensis*(11)	*Rummeliibacillus stabekisii*(1)	
	*Clostridium sporogenes*(2)	*Bacillus velezensis*(1)	*Bacillus proteolyticus* (2)	*Solibacillus isronensis*(3)	
	*Lysinibacillus macroides* (5)	*Bacillus* sp.(2)	*Bacillus siamensis*(2)	*Streptomyces virginiae*(1)	
	*Lysinibacillus xylanilyticus* (3)	*Lysinibacillus fusiformis*(5)	*Paenibacillus* sp.(1)	*Streptomyces gardneri*(1)	
	*Lysinibacillus mangiferihumi* (1)	*Lysinibacillus macroides*(8)	*Domibacillus tundrae* (1)	*Achromobacter insolitus*(1)	
	*Lysinibacillus* sp.(3)	*Lysinibacillus xylanilyticus*(2)	*Gracilibacillus marinus* (1)		
	*Savagea faecisuis* (1)	*Lysinibacillus sphaericus*(3)	*Solibacillus isronensis* (6)		
	*Rummeliibacillus stabekisii*(1)	*Lysinibacillus odyssey*(1)	*Solibacillus* sp. (2)		
	*Solibacillus isronensis*(4)	*Solibacillus isronensis*(22)			
	*Sporosarcina koreensis*(3)	*Rummeliibacillus stabekisii*(2)			
	*Sporosarcina luteola*(2)	*Staphylococcus epidermidis*(1)			
	*Staphylococcus epidermidis*(1)				

## Discussion

### OM and irrigation water contained considerable amounts of ARGs

Feng et al. (2015) has argued and illustrated that OM fertilization is more sustainable than NPK, and can boost nutrient levels in arable soils to adequate levels. The nutrient-levels (including organic C, available N, available P, and available K) at the Fengqiu fieldsite indeed all responded positively to OM fertilization (Table S2). The composition of OM is different from that of animal manure; it consisted of wheat straw, soybean oil cake, and cotton cake; these materials were machine ground into 3–5 mm sized particles, mixed thoroughly with a limited amount of water and composted for 2 months (Meng et al., 2005). Therefore, OM is intrinsically unlikely to contain bioactive compounds like antibiotics or heavy metals that are common in animal manure and may select for antibiotic resistance (Berg et al., 2010; Wu et al., 2010). We recently quantified these ARGs in pig manure and cow manure (Peng et al., 2017), and found that ARGs in OM were less diverse and less abundant, and that most of them are common in the environment. The ARGs in OM would be randomly carried by microbes that inhabit the antibiotic free ecosystem. There are several possibilities why this was not the case, e.g., (i) OM was polluted by antibiotics like tetracycline or related compounds, (ii) The composting process may enrich some microbes that produce the antibiotics. e.g., Actinobacteria and Fungi. etc., (iii) OM was colonized by bacteria (*Firmicutes*) carrying ARGs during the storage of the ingredients or proliferating during the composting procedure, resulting in the abundance of ARGs in OM (Table 1).

Irrigation water also contained considerable amounts of ARGs, of which the most abundant ARGs are *erm*(C), *sul2, sul1*, and *tet*(G) (Table 1). In this long term-fertilizer experiment, the irrigation water was ground water which was always pumped from a well; yet this irrigation water contained considerable amounts of ARGs. ARGs and ARB have been detected extensively in natural environments, such as surface rivers (Luo et al., 2011), groundwater (Koike et al., 2007; Böckelmann et al., 2009) and sediments (Devarajan et al., 2015). Even in the product water of some drinking water treatment plants (DWTPs), many ARGs have been detected (Xi et al., 2009), with *sul1, sul2, tet*(C), and *tet*(G) the four most abundant ARGs in DWTPs (Guo et al., 2014). The relative abundance of these ARGs was more than one order of magnitude higher than in the ground water of the present study. Considering that a much higher *tet*(L) gene abundance was detectable in the OM, it is possible that the irrigation water in this study was not the primary source for *tet*(L).

### Application of OM markedly increased the abundance of *tet*(L) and *intI1*

*Tet*(L) was markedly higher in OM soils than in NPK and CK soils, and this difference was consistent over 20 years (Figure 3). We speculate that there are two reasons for the difference observed. First, OM is likely the source of this elevated level of *tet*(L), which is the most abundant ARG in OM (Table 1). OM might have been colonized by ARB during storage or during the composting process, and these bacteria survived in soil. These ARB may be the key species which could promote composting of OM. However, they were not proliferated or increased continuously along with repeating application of OM, so that the abundance of *tet*(L) was in general essentially constant or even gradually lower (Figure 3). Second, *tet*(L) is also present in the CK and NPK soils, so there is indeed the possibility that *tet*(L) was already in the soil and is proliferating because the organisms carrying it are favored by OM. Previous studies found that fierce microbe–microbe competition should select for increased antibiotic production and resistance (Fierer et al., 2012), and that soil carbon is positively related to ARG abundance (Wepking et al., 2017), therefore, the increase in carbon in soil fertilized with OM may lead to increased microbial competition and a subsequent increase in *tet*(L). The difference of ARG abundance between NPK and OM soils was also observed in the data of the fresh soils (Figure 1), but from the time series we now know that this is a long standing difference. In the study of Graham et al. (2016), the level of ARGs dropped dramatically in manure fertilized soils when the source of ARGs was removed. Whether *tet*(L) would disappear when we would stop adding OM, or when we would switch to sterilized OM or a virtually *tet*(L)-free OM is still unknown.

In yearly archived samples, the abundance of *tet*(L) in NPK treated soils was essentially the same as in the CK soils, just as it was in fresh soils (Figures 1, 3). Graham et al. (2016) also reported that the level of ARGs in inorganic fertilized soils was essentially constant over time, but the time series in that study were sampled less frequently. Furthermore, a weakness of that study was that there were no unfertilized controls.

*IntI1* is commonly linked to genes conferring resistance to antibiotics (Gillings et al., 2015) and may play a role in the dissemination of ARGs (Heuer and Smalla, 2007; Gillings et al., 2008; Wang et al., 2014). In this study, *intI1* was significantly positively correlated with *tet*(L) (*r* = 0.694), this may imply that OM application has increased the intrinsic potential of OM fertilized soil for HGT. When evaluating the time series, we found that the difference of *intI1* between the three soils is decreasing (Figure 4); maybe this suggests that the amount of the bacteria carrying *intI1* is decreasing in OM or *intI1* was lost by the TRB in soil. Furthermore, the time series change of *intI1* in OM soil was somewhat similar to which in NPK soil (Figure 4), *intI1* is frequently detected in diverse environments, even in drinking water also exist abundant *intI1* (Ma et al., 2017), the improvement of the irrigation water quality may be one of the factors.

Heavy metals can exert a continuous selection pressure for antibiotic resistance in agricultural soils adjacent to feedlots (Ji et al., 2012). In this study, Cu and Pb were found weakly positively correlated with *tet*(L) (*r* = 0.309 and 0.452, respectively), this result was in agreement with results of a previous study which reported that soil ARGs might associate with Cu and Zn levels in soil (Lin et al., 2016). Cu has been shown to co-select for resistance to clinically important antibiotics in microbial soil communities under field conditions (Berg et al., 2010). Knapp et al. (2011) found that many ARGs positively correlated with soil Cu levels in archived Scottish soils. Metals can coselect for ARGs because the resistance mechanisms employed by microorganisms often leads to resistance to both and the genes are located on the same mobile elements (Knapp et al., 2011; Devarajan et al., 2015).

### OM soil bacteria shared same common genotype of *tet*(L) with NPK soil and OM

*Tet*(L) is generally located on small transmissible plasmids, which on occasion become integrated into the chromosome of staphylococci or the chromosome of *Bacillus subtilis* or into larger staphylococcal plasmids (Chopra and Roberts, 2001). Results of *tet*(L) phylogenetic analysis showed that the main genotype of *tet*(L) was same in the archived soil samples treated with OM, fresh NPK soil and OM soil, fresh OM, and irrigration water. This indicated that this main genotype of *tet*(L) is common in the environment, and that it was not affected by air-drying and OM application. Although long-term application of OM increased the abundance of *tet*(L), sequences of *tet*(L) carried by bacteria in OM soil was conservative and has not been essentially changed. The low diversity of the *tet*(L) genotypes in OM and OM soil probably results from a narrow host range of the genes carried by bacterial genera, or from the refining selection of the gene function. The high microbial density and diversity, high densities of various mobile elements, and high level of tetracycline were thought to contributed to nucleotide alterations on *tet* gene fragments (Zhang and Zhang, 2011); all three or the latter two factors may not exist in OM soil, resulting in the low diversity in *tet*(L) sequences observed.

### Survival of the bacteria carrying *tet*(L) caused the increased level of *tet*(L) in OM soil

Microorganisms from animal manure have been shown to contribute considerably to the accumulation of TRB and tetracycline resistance genes in soil (Peng et al., 2016). In this study, the bacteria carrying *tet*(L) in fresh OM were identified as *Bacillus* sp., *Lysinibacillus* sp., *Solibacillus* sp*., Rummeliibacillus* sp., and *Sporosarcina* sp. They all belong to the order *Bacillales*, and were also present in OM soil except *Sporosarcina* sp. (Table 2). Therefore we speculate that many *Bacillales* from OM were co-transferred with the OM to the soil, where their presence caused an increase in the *tet*(L) level.

Furthermore, almost all the identified TRB isolates belonged to the *Firmicutes* although a few members of the *Actinobacteria* (two *Streptomyces* sp.) and *Proteobacteria* (one *Achromobacter insolitus*) were also detected. Feng et al. (2015) have shown that OM fertilization significantly increased the relative abundance of *Firmicutes*: from 5% in the CK up to 14%. *Firmicutes* was found positively correlated with ARGs (Chen et al., 2016; Peng et al., 2017), and was also previously demonstrated to contribute to the elevated abundance and enrichment of ARGs (Zhang et al., 2016). It is possible that the transfer of *Firmicutes (Bacillales)* carrying *tet*(L) from OM to soil contributed considerably to the abundance of *tet*(L) genes in OM treated soil.

Although a wide variety of genes capable of conferring antibiotic resistance has been identified in soil microorganisms, it has been found that few of these genes are shared by human pathogens; furthermore, there is limited transfer of ARGs within the soil community and from the soil to other bacteria (Forsberg et al., 2012; Sommer, 2014). In addition, mobile ARGs have been found to be significantly enriched in *Proteobacteria* (Hu et al., 2016). Although we cannot neglect that cultivation-based approaches detect only a small fraction (usually about 1% of soil bacteria) of the total bacterial community (Amann et al., 1995), this result suggests that survival of the bacteria carrying *tet*(L) is the main reason for the increased level of *tet*(L) in OM soil, although we have found no evidence that the gene is transferred to other organisms, more in depth studies are warranted.

### Molecular analysis of archived soils can provide meaningful information on the relative abundance of ARGs in soils at the time of sampling

Dried soil samples from many sources have been stored in archives world-wide over the years (Dolfing and Feng, 2015). Previous data had shown that DNA can be effectively obtained from dried archive soils (Dolfing et al., 2004; Knapp et al., 2010, 2011; Graham et al., 2016), but the effect of drying on ARG recovery was still not studied. The abundance of the 16S rRNA gene was not obviously changed in OM soils after these soils were air-dried, while they were significantly reduced in NPK and CK soils. In the archived soil, the abundance of 16S rRNA gene fluctuated more gently in OM than in NPK and CK (Figure S3). These results were in accordance with that of Clark and Hirsch (2008); these authors found that DNA yields declined less and may be preserved by desiccation in the soil treated with farmyard manure than in the soil fertilized with NPK, which suggests that DNA stability and/or recovery during extraction were protected by the elevated level of soil organic matter.

In the present study, only in OM soil, the influence of air-drying on the relative abundance of *tet*(L) was not significant. This result may be caused by the different bacterial community structure in soil samples of OM soils compared to the communities in NPK and CK soils, it has reported that OM fertilization resulted in significantly more *Firmicutes*, significantly increased Bacilli abundance (*p* < 0.05) and changes in Bacilli composition (Feng et al., 2015). Furthermore, Tzeneva et al. (2009) found that soil drying and storage affected the bacteria community structure, probably some types of bacteria carried with *tet*(L) were tolerant to drought and survived, or persisted as spores (if they are predominantly *Bacilli*), results in this study supported this speculation: 63.6% of the isolated TRB carrying *tet*(L) belonged to *Bacilli* in fresh OM soil from 2011, while it's 98.18% in the archived OM soil from 2011, and 100% in the archived OM soil from 1989 (Table 2). Although air-drying well affected the recovery of the 16S rRNA gene in NPK and CK soil samples, the relative abundance of *tet*(L) was not reduced among all three fertilization treatments. Therefore, analyzing the abundance of *tet*(L) in archived soils by normalizing to 16S rRNA genes can be reliable: while absolute numbers are elusive, relative numbers, and consequently comparisons between different time series appear sound.

## Conclusion

In this study, we assessed the abundance of 15 ARGs and *intI1* in fresh OM soil and NPK for 22 years, and found that the abundance of *tet*(L) was noticeably increased by application of OM. We then used yearly collected archived soils to assess time series change of the *tet*(L) and *intI1* in OM, NPK, and CK soils over the past 22 years. Results indicate that *tet*(L) and *intI1* were markedly higher in OM soils than in NPK and CK soils, and that this difference was consistent over 20 years. Despite this unwanted phenomenon, our results provide cause for optimism: the normalized *tet*(L) content in the three plots is essentially constant over time. Furthermore, in OM soil, not only *tet*(L) did not increase over time (in spite of the annual dose of *tet*(L) supplied via the addition of OM), the level of *intI1* in OM soil was even decreasing in the time series, even though *intI1* was significantly and positively correlated to *tet*(L). In addition, the main genotyope of *tet*(L) in the archived OM soils, in fresh NPK soil and OM soil, in fresh OM, as well as in irrigation water was always the same, and *tet*(L) was always associated with the same culturable organisms in OM and OM soils. The increase of *tet*(L) in OM soils was a corollary result accompanied by an increase in *Firmicutes (Bacillales)*. Application of OM has previously been found to push *B. asahii* to the fore in alkaline soils, which subsequently played a key role in increased crop yield and soil fertility. Although we here report that application of OM also increased the relative abundance of *tet*(L), OM application may not further the risk of *tet*(L) dissemination, because the abundance of *tet*(L) in OM soils was essentially constant and, importantly, was always associated with the same genotype and the same organisms as in the OM itself. This result lends further support for the use of OM to maintain soil fertility.

## Author contributions

JD, YF, and XL initiated the project. YF, YW, and XL supplied soil materials and reagents. JD contributed his scientific advice during the work and MS revision. SP performed the experiments, analyzed the data, and wrote the paper. All authors reviewed the manuscript.

### Conflict of interest statement

The authors declare that the research was conducted in the absence of any commercial or financial relationships that could be construed as a potential conflict of interest.
