# Supplementary material for: Enrichment of the Antibiotic Resistance Gene tet(L) in an Alkaline Soil Fertilized With Plant Derived Organic Manure
Source: Front Microbiol. 2018 May 31;9:1140. doi: 10.3389/fmicb.2018.01140 (PMC5990627; doi:10.3389/fmicb.2018.01140)
Supplement: Supplementary file 1 [file Table_1.doc]

**Enrichment of the antibiotic resistance gene *tet*(L) in an alkaline soil fertilized with plant derived organic manure**

Shuang Peng ^1,2^, Jan Dolfing ^3^, Youzhi Feng ^1^, Yiming Wang ^1^, Xiangui Lin ^1^,*[[1]](#footnote-2)

1 Key Laboratory of Soil Environment and Pollution Remediation, Institute of Soil Science, Chinese Academy of Sciences, Nanjing 210008, China

2 College of Environment and Ecology, Jiangsu Open University, Nanjing 210017, Jiangsu, China

3 School of Engineering, Newcastle University, Newcastle NE1 7RU, UK.

**Table S1** PCR primers used in this study.

| Primer | Sequence (5′–3′) | Amplicon size (bp) | Annealing  temperature (°C) | Reference |
| --- | --- | --- | --- | --- |
| *tetB*(P)-FW  *tetB*(P)-RV | AAAACTTATTATATTATAGTG  TGGAGTATCAATAATATTCAC | 169 | 46 | Aminov et al., 2001 |
| *tet*(M)-FW  *tet*(M)-RV | ACAGAAAGCTTATTATATAAC  TGGCGTGTCTATGATGTTCAC | 171 | 55 |
| *tet*(O)-FW  *tet*(O)-RV | TACGGARAGTTTATTGTATACC  TGGCGTATCTATAATGTTGAC | 171 | 60 |
| *tet*(W)-FW  *tet*(W)-RV | GAGAGCCTGCTATATGCCAGC  GGGCGTATCCACAATGTTAAC | 168 | 64 |
| *tet*(Z)-FW  *tet*(Z)-RV | CCTTCTCGACCAGGTCGG  ACCCACAGCGTGTCCGTC | 204 | 61 | Aminov et al., 2002 |
| *tet*(G)-FW  *tet*(G)-RV | GCTCGGTGGTATCTCTGCTC  AGCAACAGAATCGGGAACAC | 468 | 55 | Ng et al., 2001 |
| *tet*(L)-FW  *tet*(L)-RV | GGTTTTGAAYGTYTCATTACCTGAT GATAGCTTTCCATATASAGCTGTTCC | 126 | 60 | You et al., 2012 |
| *erm*(B)-F | AAAACTTACCCGCCATACCA | 151 | 60 | Knapp et al., 2010 |
| *erm*(B)-R | TTTGGCGTGTTTCATTGCTT |
| *erm*(C)-F | GAAATCGGCTCAGGAAAAGG | 294 | 60 |
| *erm*(C)-R | TAGCAAACCCGTATTCCACG |
| *erm*(F)-F | TCGTTTTACGGGTCAGCACTT | 194 | 60 |
| *erm*(F)-R | CAACCAAAGCTGTGTCGTTT |
| *bla*_TEM_-F | GCKGCCAACTTACTTCTGACAACG | 247 | 55 | Devarajan et al., 2015 |
| *bla*_TEM_-R | CTTTATCCGCCTCCATCCAGTCTA |
| *bla*_CTX-M_-F | ATTCCRGGCGAYCCGCGTGATACC | 227 | 62 |
| *bla*_CTX-M_-R | ACCGCGATATCGTTGGTGGTGCCAT |
| *sul1*-F | CACCGGAAACATCGCTGCA | 158 | 60 | Chen and Zhang, 2013 |
| *sul1*-R | AAGTTCCGCCGCAAGGCT |
| *sul2*-F | TCCGGTGGAGGCCGGTATATGG | 191 | 60.8 | Pei et al., 2006 |
| *sul2*-R | CGGGAATGCCATCTGCCTTGAG |
| *sul3*-F | TCCGTTCAGCGAATTGGTGCAG | 128 | 60 |
| *sul3*-R | TTCGTTCACGCCTTACACCAGC |
| *intI1*-F | GCCTTGATGTTACCCGAGAG | 196 | 60 | Barraud et al., 2010 |
| *intI1*-R | GATCGGTCGAATGCGTGT |
| *intI2*-F | TGCTTTTCCCACCCTTACC | 195 | 60 |
| *intI2*-R | GACGGCTACCCTCTGTTATCTC |

Aminov, R. I., Garrigues-Jeanjean, N., Mackie, R. I. (2001). Molecular ecology of tetracycline resistance: development and validation of primers for detection of tetracycline resistance genes encoding ribosomal protection proteins. *Appl. Environ. Microbiol.* 67(1), 22-32.

Aminov, R. I., Chee-Sanford, J. C., Garrigues, N., Teferedegne, B., Krapac, I. J., White, B. A., Mackie, R. I. (2002). Development, Validation, and Application of PCR Primers for Detection of Tetracycline Efflux Genes of Gram-Negative Bacteria. *Appl. Environ. Microbiol.* 68(4), 1786-1793.

Barraud, O., Baclet, M. C., Denis, F., Ploy, M. C. (2010). Quantitative multiplex real-time PCR for detecting class 1, 2 and 3 integrons. *J. Antimicrob. Chemother.* 65(8), 1642-1645.

Chen, H., Zhang, M. (2013). Effects of advanced treatment systems on the removal of antibiotic resistance genes in wastewater treatment plants from Hangzhou, China. *Environ. Sci. Technol.* 47(15), 8157-8163.

Devarajan, N., Laffite, A., Graham, N. D., Meijer, M., Prabakar, K., Mubedi, J. I., et al. (2015). Accumulation of clinically relevant antibiotic-resistance genes, bacterial load, and metals in freshwater lake sediments in central europe. *Environ. Sci. Technol.* 49, 6528-6537.

Pei, R., Kim, S. C., Carlson, K. H., Pruden, A. (2006). Effect of river landscape on the sediment concentrations of antibiotics and corresponding antibiotic resistance genes (ARG). *Water Res.* 40(12), 2427-2435.

Knapp, C. W., Dolfing, J., Ehlert, P. A., Graham, D. W. (2010). Evidence of Increasing Antibiotic Resistance Gene Abundances in Archived Soils since 1940. *Environ. Sci. Technol.* 44, 580-587

Ng, L. K., Martin, I., Alfa, M., Mulvey, M. (2001). Multiplex PCR for the detection of tetracycline resistant genes. *Molecular and Cellular Probes* 15, 209-215.

You, Y., Hilpert, M., Ward, M. J. (2012). Detection of a common and persistent tet(L)-carrying plasmid in chicken-waste-impacted farm soil. *Appl. Environ. Microbiol.* 78(9), 3203-3213.

**Table S2** Change in soil chemical properties under different fertilizations in 2011

| **Treatment** | **pH** | **Organic C** | **Available N** | **Available P** | **Available K** |
| --- | --- | --- | --- | --- | --- |
|  |  | **(g kg^-1^)** | **(mg kg^-1^)** | **(mg kg^-1^)** | **(mg kg^-1^)** |
| Control | 8.86±0.03a | 4.06±0.27c | 4.00±0.48c | 0.87±0.23c | 96±8b |
| NPK | 8.60±0.16b | 5.56±0.53b | 4.48±0.72bc | 5.41±1.00b | 141±22a |
| OM | 8.50±0.17b | 9.59±0.52a | 6.48±1.21a | 10.22±1.52a | 161±20a |

Note: Data presented as average ± standard deviation from three replicates. The appearance of the different letter means that there has significant difference in one column. (*p*<0.05).

**Table S3** Change of the heavy metal concentrations (mg/kg) in the archived soil.

|  |  | 1989 | 1996 | 2002 | 2008 | 2011 |
| --- | --- | --- | --- | --- | --- | --- |
| Cr | CK | 59.49±2.76a | 61.89±3.01a | 62.22±1.59a | 59.27±0.27a | 64.93±5.67a |
|  | NPK | 62.11±2.63ab | 61.18±2.73b | 60.43±3.69b | 63.23±1.83ab | 67.26±3.13a |
|  | OM | 62.01±4.66ab | 58.18±6.37b | 61.72±2.47ab | 62.25±2.62ab | 67.37±2.73a |
| Zn | CK | 59.7±4.94a | 57.86±3.81a | 62.06±0.8a | 63.18±3.24a | 68.91±15.8a |
|  | NPK | 61.13±2.3a | 65.94±4.89a | 60.61±2.84a | 62.31±0.74a | 65.07±5.73a |
|  | OM | 61.19±3.4a | 57.87±7.22a | 63.62±3.61a | 67.69±4.36a | 66.26±6.84a |
| Cu | CK | 22.07±0.68a | 21.66±1.34a | 22.13±1.35a | 22.51±1.98a | 20.64±1.13a |
|  | NPK | 24.12±0.35a | 22.82±1.26ab | 21.95±1.55b | 22.37±0.4ab | 21.82±1.3b |
|  | OM | 23.86±1.69a | 21.25±1.97a | 23.22±1.29a | 26.46±8.59a | 23.41±1.96a |
| Ni | CK | 24.29±1.42a | 24.63±1.72a | 24.03±0.97a | 24.33±1.27a | 24.22±1.04a |
|  | NPK | 25.07±0.69a | 25.38±1.45a | 24.24±1.46a | 25.06±0.78a | 25.58±1.21a |
|  | OM | 25.34±1.24a | 24.01±2.7a | 24.82±1.14a | 24.59±0.95a | 25.6±1.76a |
| Pb | CK | 18.13±0.58b | 18.81±0.68ab | 18.5±0.12ab | 18.76±0.77ab | 20.05±1.67a |
|  | NPK | 18.71±1.13a | 18.92±0.63a | 18.97±0.93a | 19.73±0.31a | 19.82±0.55a |
|  | OM | 19.47±0.97ab | 17.78±1.79b | 23.41±5.86a | 19.09±0.84ab | 19.52±1.02ab |
| Cd | CK | 0.07±0.02b | 0.12±0.03a | 0.1±0.01ab | 0.11±0.02ab | 0.1±0.01ab |
|  | NPK | 0.09±0.03b | 0.15±0.04a | 0.1±0.01ab | 0.12±0.02ab | 0.15±0.02a |
|  | OM | 0.09±0.04a | 0.12±0.04a | 0.12±0.04a | 0.11±0.01a | 0.14±0.01a |
| As | CK | 8.63±0.47a | 8.88±0.46a | 8.97±0.34a | 9.15±0.48a | 9.04±0.48a |
|  | NPK | 8.87±0.12c | 9.08±0.16bc | 8.94±0.27c | 9.51±0.32ab | 9.58±0.27a |
|  | OM | 8.84±0.3a | 8.97±0.18a | 8.93±0.09a | 9.14±0.36a | 9.15±0.23a |
| Hg | CK | 0.01±0b | 0.02±0b | 0.03±0.01a | 0.02±0b | 0.02±0b |
|  | NPK | 0.01±0b | 0.03±0.02a | 0.04±0a | 0.04±0a | 0.04±0a |
|  | OM | 0.01±0b | 0.02±0b | 0.03±0a | 0.03±0a | 0.03±0.01a |

Different lowercase letters indicate significant differences among soil samples collected in different year (*P* < 0.05).

**Table S4** Correlation analysis of the relative abundance of *tet*(L) and heavy metal concentrations in archived soils

|  | Cr | Ni | Cu | Zn | Cd | Pb | As | Hg |
| --- | --- | --- | --- | --- | --- | --- | --- | --- |
| *tet*(L) | -0.05 | -0.016 | 0.309* | 0.048 | 0.095 | 0.452** | 0 | 0.091 |

In each cell, the value indicates the Pearson correlation coefficient (r), and the asterisk behind the value indicate statistical significance (*, *P* < 0.05; **, *P*<0.01).


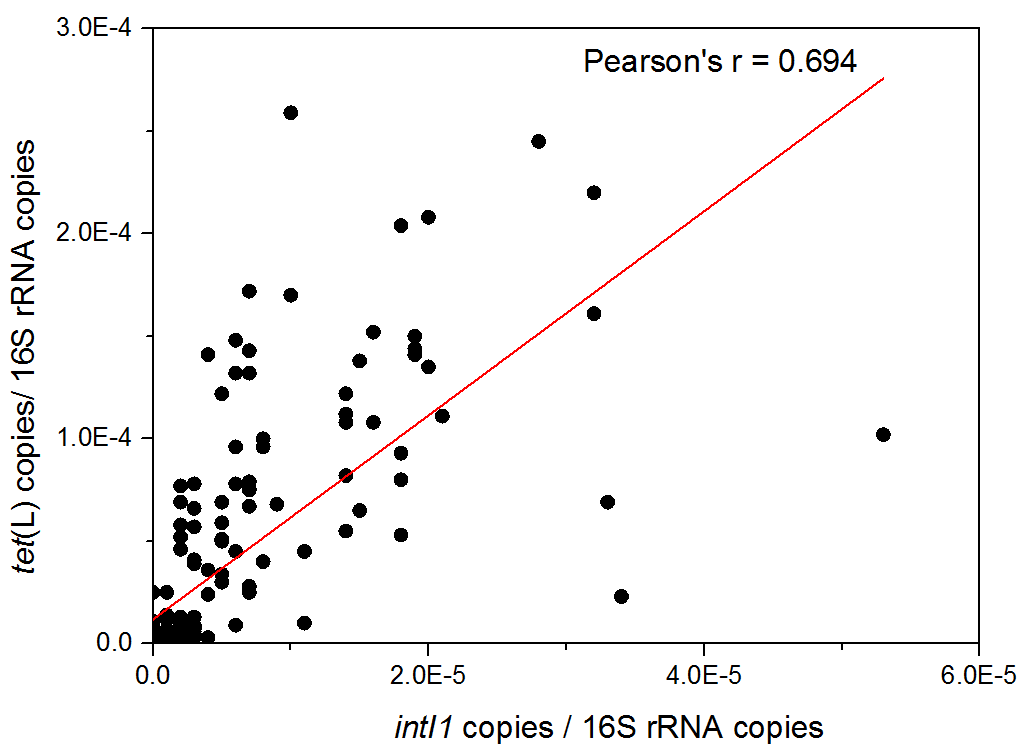


**Figure S1** Correlation between the relative abundance of *tet*(L) and that of *intI1* in the soils.


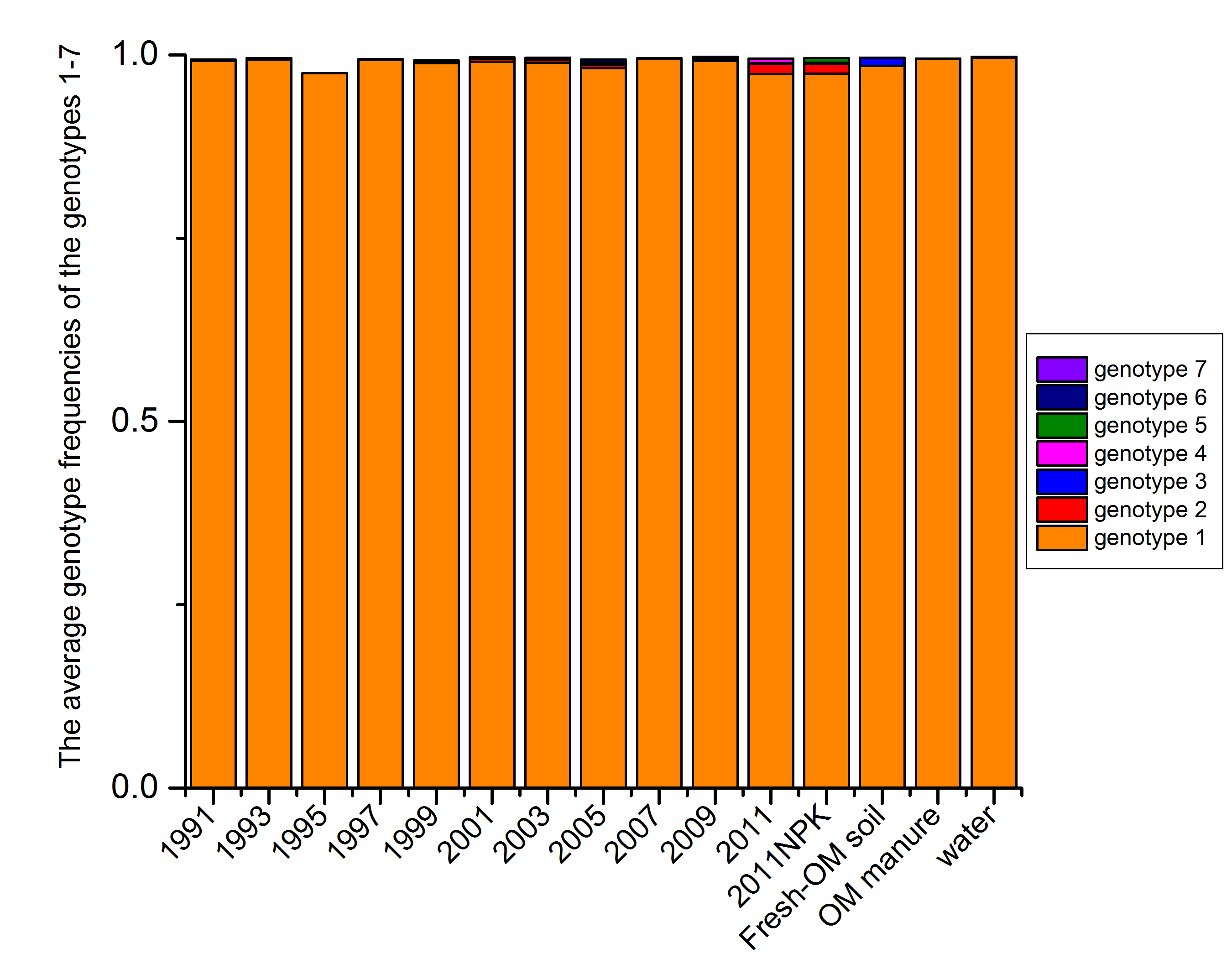


**Figure S2** The average frequencies of *tet*(L) genotypes 1- 7 in different samples.


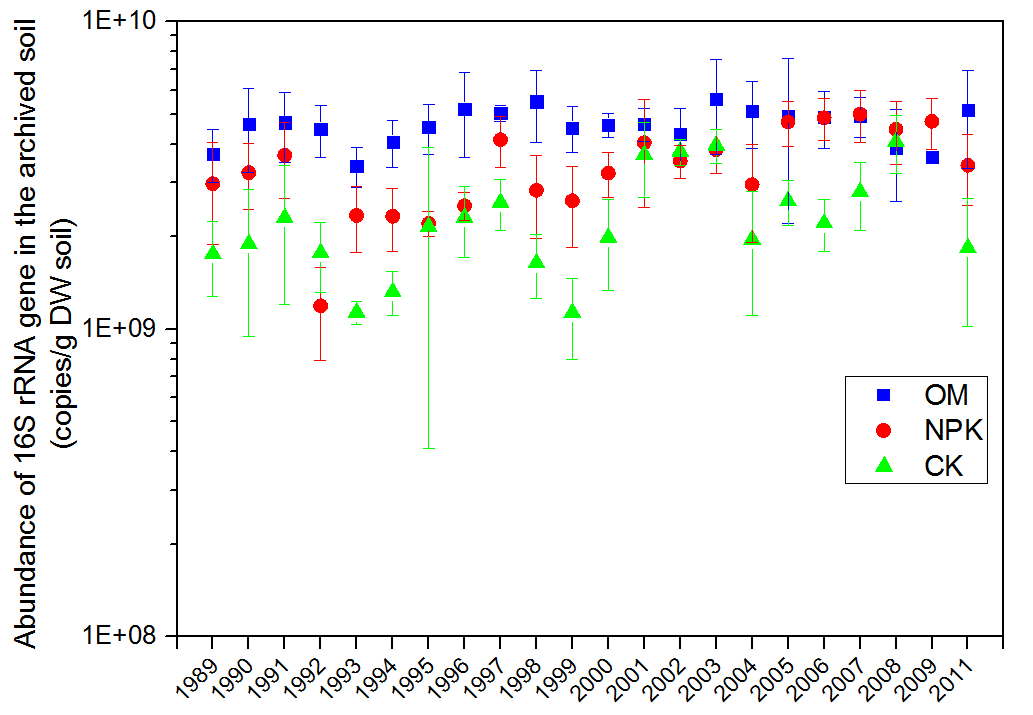


**Figure S3** Changes in the abundance of the 16S rRNA gene in different fertilizer treated archived soils.

1. ^*^ Corresponding author. Tel.: +86 25 8688 1589; Fax: +86 25 8688 1000; E-mail address: [xglin@issas.ac.cn](mailto:xglin@issas.ac.cn) (X. Lin). [↑](#footnote-ref-2)
